# Association of *DARS* gene polymorphisms with the risk of isolated ventricular septal defects in the Chinese Han population

**DOI:** 10.1186/s13052-016-0311-2

**Published:** 2016-11-21

**Authors:** Yu Feng, Runsen Chen, Xuming Mo

**Affiliations:** Department of Cardiothoracic Surgery, Children’s Hospital of Nanjing Medical University, Nanjing, Jiangsu 210000 China

**Keywords:** Ventricular septal defect, Aminoacyl-tRNA synthetases, Association study, Polymorphism

## Abstract

**Background:**

Ventricular septal defects (VSD) are the most common subtype of congenital heart defects (CHD) and are estimated to account for 20 to 30% of all cases of CHD. The etiology of isolated VSD remains poorly understood. Eight core aminoacyl-tRNA synthetases (ARSs) (EPRS, MARS, QARS, RARS, IARS, LARS, KARS, and DARS) combine with three nonenzymatic components to form a complex known as the multisynthetase complex (MSC). Four single nucleotide polymorphisms (SNPs) in EPRS have been reported to be associated with risks of CHD in Chinese populations.

**Methods:**

In this study, we hypothesize that SNPs of the DARS gene might influence susceptibility to sporadic isolated VSD. Therefore, we conducted a case-control study of 841 patients with isolated VSD and 2953 non-CHD controls from the Chinese Han population to evaluate how 4 potentially functional SNPs within the DARS gene were associated with the risk of VSD.

**Results:**

We observed that the risk of VSD was significantly associated with rs2164331 [G/A; odds ratio (OR) = 0.78, 95% confidence interval (CI) = 0.69-0.91; P = 3.17 × 10^-3^], rs6738266 [G/A; OR = 1.17, 95% CI = 1.05-1.29, P = 1.83 × 10^-3^], and rs309143 [G/A; OR = 1.09, 95% CI = 1.01-1.17; P = 3.12 × 10^-2^]. Additionally, compared with individuals with 0-2 risk alleles, individuals carrying 3, 4, and 5 or more risk alleles had 1.01-, 1.22- and 1.46-fold greater risks of VSD, respectively. These findings revealed a significant dose-response effect for VSD risk among individuals carrying different numbers of risk alleles (P_trend_ = 6.37 × 10^-4^).

**Conclusions:**

These findings indicate that genetic variants of the *DARS* gene may influence individual susceptibility to isolated VSD in the Chinese Han population.

## Background

Congenital heart defects (CHD) are the most common major human birth malformation, affecting approximately 8 per 1,000 live births [1, 2]. CHD are associated with significant morbidity and mortality and are second only to infectious diseases with respect to contributing to infant mortality rate [3]. In China, the overall mortality rate of CHD increased from 141 per 10,000,000 person-years in 2003 to 229 per 10,000,000 person-years in 2010, a 62.4% relative increase [4]. With the advances in surgical techniques, the prognosis of children with complicated and uncomplicated CHDs continues to improve, but the reported incidence remains unchanged [5]. Ventricular septal defects (VSD) are the most common subtype of CHD and are estimated to account for 20 to 30% of all CHD [6]. The majority of VSD (~80%) occur as sporadic events, although certain VSD affect multiple family members [7].

The etiology of VSD is complex and possibly includes the interaction of inherited factors and environmental exposures [8]. A multitude of research studies have identified both chromosomal abnormality and gene mutations as causation for the syndromic heart malfunction [9]. However, the origin of isolated VSD is waiting to be uncovered further [10].

Over the past decades, plenty of genes have been identified as candidates to be responsible for VSD [9]. However, aminoacyl-tRNA synthetases (ARSs) that seemed to be in charge of only cellular protein synthesis were overlooked [11, 12]. ARSs catalyze the attachment of amino acids to their cognate tRNAs with high fidelity [13, 14]. Recent research has shown that eukaryote ARSs, distinguished from their prokaryotic counterparts, have additional domains and motifs such as glutathione S-transferase (GST), WHEP domains, leucine zipper domains, and α-helicalappendices that function beyond translation [15] and may link with a variety of human diseases, such as cancer, neuronal pathologies, autoimmune disorders, and disrupted metabolic conditions [16]. Recently, the nontranslational functions of vertebrate ARSs have been associated with cytoplasmic forms and nuclear and secreted extracellular forms that impact cardiovascular development pathways [17].

Eight core aminoacyl-tRNA synthetases (ARSs), bifunctional glutamyl-prolyl-tRNA synthetase (*EPRS*), isoleucyl-tRNA synthetase (*IRS*), leucyl-tRNA synthetase (*LRS*), methionyl-tRNA synthetase (*MRS*), glutaminyl-tRNA synthetase (*QRS*), lysyl-tRNA synthetase (*KRS*), aspartyl-tRNA synthetase (*DARS*), and arginyl-tRNA synthetase (*RRS*), form a macromolecular protein complex with three auxiliary factors, designated ARS-interacting multifunctional protein 1 (AIMP1), AIMP2 and AIMP3. This complex is known as the multisynthetase complex (MSC). The MSC may act as a depot for ARSs, which could be subsequently released from the macromolecular complexes to participate in auxiliary tasks beyond translation [18], generate a channel for the delivery of tRNAs and help the proofreading of newly synthesized nuclear tRNAs in the nucleus. Recently, four single nucleotide polymorphisms (SNPs) in *EPRS* have been reported to be associated with risks of congenital heart disease in Chinese populations [19].

To determine the effects of genetic variants of the *DARS* gene on the development of isolated VSD, we conducted a case-control study that investigated the genotype frequency distribution of these 4 potentially functional polymorphisms.

## Methods

### Ethics statement

This study was approved by the institutional review board (IRB) of Nanjing Medical University and adhered to the tenets of the Declaration of Helsinki. The design and performance of the current study involving human subjects were clearly described in a research protocol. All participants and/or their parents were volunteered and completed an informed consent in writing before taking part in this research.

### Subjects

The case-control analysis included 841 affected children with sporadic isolated VSD and 2953 unrelated non-CHD controls. Subjects for this study were consecutively recruited from the Affiliated Nanjing Children’s Hospital of Nanjing Medical University, China, from March 2009 to May 2016. All isolated VSD patients were diagnosed based on echocardiography, with some diagnoses further confirmed by cardiac catheterization and/or surgery diagnosed based on surgery. Cases that had multiple major developmental anomalies, including developmental syndromes, or known chromosomal abnormalities were excluded. The exclusion criteria also included a positive family history of CHD in a first-degree relative (parents, siblings and children), phenylketonuria, maternal diabetes mellitus, maternal teratogen exposure (e.g., pesticides and organic solvents), and maternal therapeutic drug exposure during the intrauterine period. Controls were non-CHD outpatients from the same geographic areas and same time period. For each participant, approximately 4 ml of whole blood was obtained to extract genomic DNA for genotyping analysis.

### SNP selection and Sanger sequencing

For the *DARS* gene, we first used the public HapMap single nucleotide polymorphism (SNP) database (phase II + III, Feb 09, on NCBI B36 assembly and dbSNP b126) to search for SNPs localized within gene regions with MAF ≥ 0.05 in the Chinese Han population. Subsequently, a web-based analysis tool was used to predict the function of these SNPs (https://snpinfo.niehs.nih.gov/snpinfo/snpfunc.php). Finally, a total of 8 potentially functional SNPs were selected. We next conducted linkage disequilibrium (LD) analysis using Haploview 4.2 software (Broad Institute of MIT and Harvard, Boston, MA), and 4 SNPs were selected in cases involving multiple SNPs in the same haplotype block (*r* ^*2*^ > 0.8). Four SNPs (rs2164331, rs6738266, rs309142 and rs309143) remained.

Primers were designed using Primer-BLAST. Variant-containing PCR products were directly sequenced using a nested primer (ACGT, Inc., Germantown, MD). Sequence data were analyzed using FinchTV.

### Statistical analyses

The differences between the VSD patients and control subjects were evaluated in the distributions of selected variables, and frequencies of genotypes of the four polymorphisms using Student’s *t*-test (for continuous variables) or the Chi-square test (for categorical variables). The Chi-square test determined the Hardy-Weinberg equilibrium of the genotype distribution of polymorphisms in the control group. LD between SNPs was evaluated using Haploview 4.2. Odds ratios (ORs) and 95% confidence intervals (CIs) were estimated by logistic regression analyses in the additive model to estimate the associations between the variants genotypes and risk of VSD. All statistical analyses were performed using the Statistical Analysis System software (v.9.1.3; SAS Institute, Cary, NC, USA). All tests were two-sided, and *P* < 0.05 was considered significant.

## Results

We systematically investigated the association of potentially functional SNPs with VSD susceptibility in 841 cases and 2953 controls in a Chinese population. There were no statistically significant differences between the cases and controls with respect age and gender distributions (*P* = 0.544 and *P* = 0.77, respectively) (Table 1).

**Table 1:** Distributions of select variables in VSD cases and non-CHD controls

Variables	Cases(*N* = 841)	Controls(*N* = 2953)	*P*
Age, years (mean ± s.d.)	1.51 ± 2.23	1.46 ± 2.07	0.544
Gender			
Male	409(48.63%)	1453(49.20%)	0.77
Female	432(51.37%)	1500(50.80%)	

The genotype distributions of the four SNPs and their associations with VSD risk are summarized in Table 2. The observed genotype frequencies of these SNPs were in agreement with Hardy-Weinberg equilibrium in the controls (*P* values between 0.37 and 0.52). Among the four SNPs, rs2164331, rs6738266, and rs309143 were significantly associated with VSD risk based on logistic regression analysis in the additive model. The A allele of rs2164331 was associated with a decreased risk of VSD [additive model: OR = 0.78, 95% CI = 0.69-0.91, *P* = 3.17 × 10^-3^]; however, the A allele of rs6738266 and the G allele of rs309143 were associated with an increased risk of VSD (OR = 1.17, 95% CI = 1.05-1.29, *P* = 2.83 × 10^-3^, and OR = 1.09, 95% CI = 1.01-1.17, *P* = 3.12 × 10^-2^, respectively). Additionally, we performed haplotype analysis. As indicated in Table 3, the haplotype “GAG” (the combination of the risk alleles of the three SNPs) was associated with an increased risk of VSD, whereas the protective allele combination “AGA” was associated with a decreased risk of VSD.

**Table 2:** Summary of associations between 4 SNPs of DARS gene with VSD

Chr.	Gene	SNP	Allele^a^	MAF^b^		HWE^c^	Additive model	
(cytoband)				Cases	Controls		OR (95% CI)	*P*
1q41	*DARS*	rs2164331	G/A	0.22	0.26	0.37	*0.78 (0.69-0.91)*	*3.17 × 10* ^*-3*^
		rs6738266	G/A	0.06	0.03	0.52	*1.17 (1.05-1.29)*	*2.83 × 10* ^*-3*^
		rs309143	A/G	0.23	0.22	0.41	*1.09 (1.01-1.17)*	*3.12 × 10* ^*-2*^
		rs309142	A/G	0.43	0.43	0.29	1.01 (0.91-1.12)	9.11 × 10-1

^a^Major/minor allele

^b^Minor allele frequency among cases/controls

^c^Hardy-Weinberg equilibrium test among controls

**Table 3:** The haplotypic association of the four SNPs of the *DARS* gene with VSD

Haplotype^a^	Case (%)	Control (%)	OR (95% CI)	*P*
GGA	712(42.33)	2551(43.19)	1.00(referent)	
GGG	562(33.41)	1893(32.05)	1.06(0.94-1.21)	3.35 × 10^-1^
AAG	94(5.59)	289(4.89)	1.17(0.91-1.49)	2.24 × 10^-1^
GAG	90(5.35)	248(4.20)	*1.30(1.01-1.68)*	*4.30 × 10* ^*-2*^
AGA	140(8.32)	681(11.53)	*0.74(0.60-0.90)*	*3.00 × 10* ^*-3*^
Others	84(4.99)	244(4.13)	1.23(0.95-1.60)	1.15 × 10^-1^

^a^SNP order: rs2164331, rs6738266, rs309143

We also conducted a combined analysis of the four promising SNPs to test their joint effects on VSD risk. There was a significant dose-response effect with respect to VSD risk among individuals carrying different numbers of risk alleles (*P*_trend_ = 6.37 × 10^-4^). Compared with individuals with 0-2 risk alleles, those carrying 3, 4 or 5 or more risk alleles had 1.01-, 1.22- or 1.46-fold greater risks of VSD, respectively (Table 4).

**Table 4:** Combined effects of rs2164331, rs6738266, and rs309143 on isolated VSD

Number of risk alleles^a^	Case (%)	Control (%)	OR (95% CI)^b^	*P* ^*b*^
0-2	231 (27.47)	949 (32.14)	1.00	
3	68 (8.08)	276 (9.35)	1.01(0.75-1.37)	9.37 × 10^-1^
4	361 (42.93)	1219 (41.28)	1.22 (1.01-1.47)	3.8 × 10^-2^
≥5	181(21.52)	509(17.24)	1.46(1.17-1.83)	1.0 × 10-3
Trend				6.37 × 10^-4^

^a^rs2164331 A, rs6738266 A, rs309143 G were assumed as risk alleles

^b^Calculated by additive model

## Discussion

In this study, we systematically investigated how four potentially functional SNPs in one ARS-coding gene, *DARS*, were associated with isolated VSD susceptibility in 841 cases and 2953 controls from a Chinese population. We observed that three SNPs (rs2164331, rs6738266, rs309143) in the *DARS* gene were significantly associated with the risk of VSD, which was remarkably elevated in individuals who carried more risk alleles. Although VSD are the most common congenital heart malfunctions, the pathogenesis of these defects is poorly understood, particularly for isolated VSD. Based on prior research, SNPs in *EPRS*, another core coding gene in the MSC, may modulate the process of CHD in the Chinese Han population [19]. In the present study, we provided evidence that SNPs in *DARS* might play important roles in isolated VSD.

*DARS* (MIM 603084, RefSeq accession number NM_001349.2) encodes an aspartyl-tRNA synthetase (AspRS) [20]. Mutations in this gene have been found in patients who exhibit hypomyelination with brainstem and spinal cord involvement and leg spasticity [21, 22]. Homsy et al. [23] used exome sequencing analysis in 1,213 CHD parent-offspring trios to identify several protein-damaging *de novo* mutations, particularly in genes that are highly expressed in the developing heart and brain. They identified mutations that accounted for 20% of patients with CHD, neurodevelopmental disabilities (NDD) and other congenital anomalies (CA), suggesting that CHD, NDD, and other CA may share genetic characteristics. Based on its expressed sequence tag (EST) profile in the public database UniGene (http://www.ncbi.nlm.nih.gov/UniGene), the *DARS* gene is expressed in human heart tissue at a level of 147 transcripts per million.

Transcription factors are known to play a fundamental role in all stages of heart development, including cardiac lineage determination, chamber formation, valvulogenesis and septation [24]. All three of the significant SNPs are located in transcription factor binding sites and could therefore affect binding between transcription factors and DARS. According to the online tool SNPinfo, the SNP rs6738266 is an exonic splicing enhancer (ESE) [25]. The mutation of ESE motifs significantly contributes to genetic disorders and certain cancers. Simple point mutations in ESEs can inhibit affinity for splicing factors and alter alternative splicing, leading to altered mRNA sequences and protein translation [26].

Several limitations of the present study need to be addressed. First, we did not replicate the results in additional individuals; this may contribute to potential false positive errors. The present analysis was restricted to individuals of Chinese Han descent, and therefore, the findings may not hold true for individuals of other races and ethnicities. Additionally, the limited sample size may have contributed to the weak statistical power of this study; thus, further replication of the association signal in an independent cohort for the three SNPs would support the study’s conclusions. Therefore, the results are required to be further replicated by well-designed studies in additional large-scale Chinese Han populations.

## Conclusion

In conclusion, we conducted a case-control study to investigate the roles of genetic variants in one ARS-coding gene, *DARS*, in the development of isolated VSD in a Chinese population. We observed that three SNPs (rs2164331, rs6738266, and rs309143) may confer susceptibility to sporadic isolated VSD and that risk significantly increased with the number of risk alleles. However, further studies involving functional evaluations are warranted to elucidate the potential biological mechanisms of these polymorphisms in the development of VSD.

## Abbreviations

AIMP1: ARS-interacting multifunctional protein 1
ARSs: Aminoacyl-tRNA synthetases
CHDs: Congenital heart defects
DARS: Aspartyl-tRNA synthetase
EPRS: Bifunctional glutamyl-prolyl-tRNA synthetase
GST: Glutathione S-transferase
IRS: Isoleucyl-tRNA synthetase
KRS: Lysyl-tRNA synthetase
LRS: Leucyl-tRNA synthetase
MRS: Methionyl-tRNA synthetase
MSC: Multisynthetase complex
QRS: Glutaminyl-tRNA synthetase
RRS: Arginyl-tRNA synthetase
SNPs: Single nucleotide polymorphisms
VSD: Ventricular septal defect
